# A Smart Card-Based Two-Factor Mutual Authentication Scheme for Efficient Deployment of an IoT-Based Telecare Medical Information System

**DOI:** 10.3390/s23125419

**Published:** 2023-06-07

**Authors:** Muhammad Asghar Khan, Hosam Alhakami, Wajdi Alhakami, Alexey V. Shvetsov, Insaf Ullah

**Affiliations:** 1 Department of Electrical Engineering, Hamdard Institute of Engineering and Technology, Hamdard University, Islamabad 44000, Pakistan; insaf.ullah@hamdard.edu.pk; 2 Department of Computer Science, College of Computer and Information Systems, Umm Al-Qura University, Makkah 21955, Saudi Arabia; hhhakam@uqu.edu.sa; 3 Department of Information Technology, College of Computers and Information Technology, Taif University, Taif 21431, Saudi Arabia; whakami@tu.edu.sa; 4 Department of Smart Technologies, Moscow Polytechnic University, St. Bolshaya Semenovskaya, 38, 107023 Moscow, Russia; a.shvetsov@vvsu.ru; 5 Faculty of Transport, North-Eastern Federal University, St. Belinsky, 58, 677000 Yakutsk, Russia

**Keywords:** internet of things, telecare medicine information system, smart card, mutual authentication, hyperelliptic curve cryptography

## Abstract

The integration of the Internet of Things (IoT) and the telecare medical information system (TMIS) enables patients to receive timely and convenient healthcare services regardless of their location or time zone. Since the Internet serves as the key hub for connection and data sharing, its open nature presents security and privacy concerns and should be considered when integrating this technology into the current global healthcare system. Cybercriminals target the TMIS because it holds a lot of sensitive patient data, including medical records, personal information, and financial information. As a result, when developing a trustworthy TMIS, strict security procedures are required to deal with these concerns. Several researchers have proposed smart card-based mutual authentication methods to prevent such security attacks, indicating that this will be the preferred method for TMIS security with the IoT. In the existing literature, such methods are typically developed using computationally expensive procedures, such as bilinear pairing, elliptic curve operations, etc., which are unsuitable for biomedical devices with limited resources. Using the concept of hyperelliptic curve cryptography (HECC), we propose a new solution: a smart card-based two-factor mutual authentication scheme. In this new scheme, HECC’s finest properties, such as compact parameters and key sizes, are utilized to enhance the real-time performance of an IoT-based TMIS system. The results of a security analysis indicate that the newly contributed scheme is resistant to a wide variety of cryptographic attacks. A comparison of computation and communication costs demonstrates that the proposed scheme is more cost-effective than existing schemes.

## 1. Introduction

The telecare medicine information system (TMIS) is an integrated network of medical equipment and sensors that provides preventative or proactive healthcare services at a low cost [1]. This technology enables physicians and patients to access health-related records via the Internet at any time and from any location [2]. Therefore, maintaining a patient’s personal medical information and providing timely medical services have become viable procedures for the modern medical industry [3]. Moreover, in today’s chaotic environment, remote system access has become an unavoidable technique that the average user utilizes. Sensors on the patient’s body transmit data to a smartphone, which then transmits the data to a health provider’s servers via the Internet. In addition to facilitating access to health-related data, this technology helps keep patients and physicians informed about environmental parameters such as patient care settings, laboratory shift schedules, treatment durations, and staff-to-patient ratios [4]. If necessary, the patient can receive first aid in the form of an ambulance before arriving at the hospital. To anticipate emergencies, the research and development (R&D) division analyzes sensor inputs for behaviour in depth.

On the one hand, this technology is deemed essential and should be incorporated into the existing global healthcare system [5,6,7]; on the other hand, the open nature of the Internet, the primary source of connectivity and data exchange, raises security and privacy concerns [8]. The leading contributors to these security and privacy concerns are as follows: (1) Medical devices and healthcare sensors are well interconnected, and some of these devices and sensors can even connect automatically due to dynamic network configuration settings. (2) An open wireless channel between health system devices and legacy systems can increase their vulnerability by granting malicious attackers unauthorized access to systems or data. (3) Unauthorized access is also crucial in the smart hospital environment, as the absence of an authorization policy could lead to unauthorized users obtaining access to a vital system via an end device. Therefore, the primary requirements for developing security and privacy for TMIS are as follows: (1) all data values must comply with semantic standards without tampering; (2) all medical services and data must be continuously accessible to the user (patient, nurse, practitioner, or provider); (3) all systems must be used only by authorized users; (4) data must be transmitted securely during all communications between communicating parties; and (5) all patients’ private information must be protected. By proposing an efficient authentication scheme, all of these security requirements can be met. Once the authentication between the user and the medical server has been validated, any authorized user will have remote access to the server’s information [9]. Practically every authentication system for remote users employs smart cards.

Researchers have developed a variety of two-factor authentication solutions to address this issue. Researchers are emphasizing the development of more secure and highly efficient remote authentication schemes that combine two factors; consequently, password-based authentication using a smart card is gaining popularity. In 1981, Lamport [10] was the first to propose a method for performing remote authentication over an unsecured public channel. Over the past three decades, many research articles on password-based authentication systems have been published. The design of these articles was founded on the article blueprint proposed by Lamport [10].

The literature extensively uses elliptic curve cryptography (ECC) to develop a cryptographic algorithm for smart card-based password authentication. The ECC procedure incurs significant computational and communication overhead. Consequently, we can use hyperelliptic curve cryptography (HECC), a refined form of ECC that maintains the same level of security despite employing shorter keys, identities, and certificates than ECC’s 160 bits [11]. In addition, the TMIS system would render HECC the best option for low-power devices. In this paper, we present a smart card and HECC-based efficient and secure two-factor authentication scheme for the TMIS. Here are some of the key contributions of this study:

- We propose an efficient and provably secure two-factor authentication scheme based on hyperelliptic curve cryptography (HECC) with a smart card-based approach;
- We use the most advantageous property of HECC, a smaller key size, to make the proposed scheme as lightweight as possible;
- The proposed authentication scheme consists of two steps: validation and verification; on the reader side, the smart card performs the first phase of authentication while the server manages the second phase.
- The proposed scheme is resistant to a variety of attacks, as demonstrated by formal and informal analyses;
- Finally, after comparing the performance of the proposed scheme with that of the existing schemes, it was found that the proposed scheme is more cost-effective than the existing schemes in terms of computation and communication costs.

The subsequent sections are structured as follows: Section 2 outlines the literature review. In Section 3 and Section 4, the network architecture of the proposed scheme is described. In Section 5, the security analysis, which includes both formal and informal analysis, is presented. Section 6 contains a performance evaluation, while Section 7 provides concluding remarks.

## 2. Literature Review

The TMIS generates the finest patient monitoring, a well-organized diagnostic process, and intensive support and care compared to conventional healthcare operations. To facilitate these highly technological adaptations, however, data leakage and privacy thievery threaten the confidentiality of patients’ personal information in the current revolution [12,13]. In recent years, numerous ECC-based authentication and key agreement techniques [14] have been presented to address these security and privacy issues. Nonetheless, many of these approaches have been analyzed and found to be vulnerable to a variety of well-known security flaws. After examining the relevant published articles, our investigation uncovered this information. In 2010, Wu et al. [15] proposed an authentication method for TMIS using smart cards based on a password. He et al. [16], however, demonstrated that the technique presented by Wu et al. [15] is vulnerable to impersonation and insider attacks. Then, in response to Wu et al.’s scheme, He et al. [16] devised a better authentication technique. Wu et al. [15] and He et al. [16] have demonstrated that they do not meet the fundamental security requirements of a two-factor authentication method [17]. Wei et al. [17] proposed an authentication method for TMIS applications and demonstrated that their proposed system satisfies all two-factor authentication scheme security requirements. Xu et al. [18] developed a computationally efficient two-factor mutual authentication technique. With the incorporation of dynamic identification, this scheme enhanced patient anonymity. The authors assert that the proposed method is significantly more secure and efficient than comparable two-factor authentication methods.

In 2014, Islam et al. [19] suggested that the method proposed by Xu et al. [18] is applicable in practice due to the absence of the following requirements: (a) Firstly, the Xu et al. [18] technique was unable to provide strong authentication throughout the entire logon and authentication phases; (b) secondly, this system does not permit the user to change his password during the password-changing phase; and (c) finally, this scheme was unable to execute the strong replay attack. Chaudhry et al. [20] came up with a revised authentication procedure to fix the vulnerability that Islam et al. [19] discovered and disclosed. Nevertheless, Qiu et al. [21] revealed that both the Chaudhry et al. [20] and the Islam et al. [19] systems are susceptible to offline password guessing, user impersonation, server assaults, and man-in-the-middle attacks. A smart card-based authentication system was presented by Qiu et al. [21] as a means of overcoming the limitations of the two previously discussed methods.

Ostad-Sharif et al. [22] introduced an ECC-based authentication and key agreement protocol for the TMIS. Kumari et al. [23] demonstrated that the approach devised by Ostad-Sharif et al. [22] is vulnerable not only to key compromise impersonation attacks but also to key compromise password guessing attacks. Using ECC and smart cards, Radhakrishnan et al. [24] proposed a two-factor authentication scheme. This procedure was created to be both efficient and secure. All of the schemes, as mentioned earlier, are either not secure, i.e., they have security issues and do not meet the security requirements, or they are computationally efficient and unsuitable for resource-constrained biomedical devices. Keeping these observations in mind and employing the HECC concept, we propose a new solution: a smart card-based two-factor mutual authentication scheme. Two stages comprise the proposed authentication scheme: validation and verification. The smart card performs the first phase of authentication on the reader side, while the server handles the second phase. Formal and informal analyses demonstrate that the proposed scheme is resilient against a variety of attacks. In addition, we employ the most advantageous feature of HECC, a smaller key size, to make the proposed scheme as lightweight as feasible.

## 3. Network Architecture

**Figure 1:**
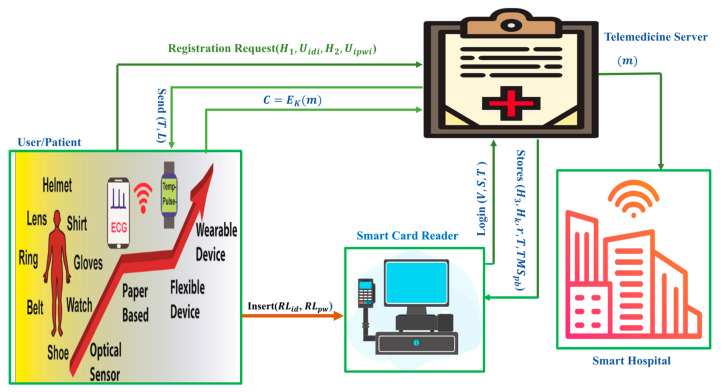
Illustration of the architecture of the TMIS system.

The architecture of the smart card-based two-factor mutual authentication scheme for efficient deployment of an IoT-based TMIS is illustrated in Figure 1. This diagram depicts numerous entities, such as the telemedicine server (TMS), the user/patient, the smart card reader, and the smart hospital. The following is an explanation of the tasks performed by the entities listed above in our proposed scheme.

User/Patient: The user/patient contains several medical sensors; further, it is responsible for inserting his real identity and actual password ($RL_{id}$, $RL_{pw}$) to the smart card, and it sends the registration request with a tuple ($H_{1},U_{idi},H_{2},U_{ipwi}$) to the telemedicine server (TMS).

Telemedicine Server (TMS): When it receives the registration request from the user/patient with ($H_{1},U_{idi},H_{2},U_{ipwi}$), it generates and stores ($H_{3},H_{k},r,T,TMS_{pb}$) into the smart card of the user/patient. Further, when it receives the login request by using (V,S,T) from the smart card reader, it generates and sends a mutual-authentication text (L,T) to the user/patient. Moreover, when it receives the ciphertext from the user/patient, it decrypts the ciphertext by using the shared secret key and delivers the plaintext to application providers in the hospital.

Smart Card Reader: This is responsible for generating login requests by using (V,S,T) and sending them to the telemedicine server (TMS). Moreover, it keeps the stored data ($H_{3},H_{k},r,T,TMS_{pb}$) secret.

Smart Hospital: It contains application providers such as nurses, doctors, and emergency services. The role of application providers is to monitor the patient’s situation through received plaintext from the telemedicine server (TMS) and then take action accordingly.

## 4. Proposed Smart Card-Based Two-Factor Mutual Authentication Scheme

The construction of the proposed scheme—a smart-card-based two-factor mutual authentication scheme—consists of the five phases as listed in [18], and Table 1 illustrates the symbols used in the proposed scheme.

1. Initialization: The telemedicine server (TMS) can select the hyperelliptic curve ($HEC_{g=2}$) with a genus equal to or less than 2 and a devisor (D) that contains 80 bits. Additionally, the finite field ($F_{d}$) has a size that is not greater than 80 bits. The TMS selects its private key from ($TMS_{pr}$) and computes its master public key using the equation $TMS_{pb}$ = $TMS_{pr}.D$. At the very end, it is possible for it to define some hash functions, such as (H), from the SHA family.
2. Key Generation: The user ($U_{i}$) selects his private key from ($U_{pr}$) and computes his public key using the equation $U_{pb}=U_{pr}.D$
3. Registrations: With the real identity of ($RL_{id}$) and password ($RL_{pw}$), the user ($U_{i}$) can perform the following steps:
  - Choose (r) from $F_{d}$ and compute $U_{idi}=E_{TMS_{pb}}\left(RL_{id},T,r\right)$;
  - Calculate $U_{ipwi}=E_{TMS_{pb}}\left(RL_{pw},T,r\right)$, $H_{1}=h\left(RL_{id},T,r\right)$;
  - Calculate $H_{2}=h\left(RL_{pw},T,r\right)$ and send ($H_{1},U_{idi},H_{2},U_{ipwi}$) to the TMS;
  When ($H_{1},U_{idi},H_{2},U_{ipwi}$) is received by the TMS, it performs the following steps:
  - It calculates $H_{3}=h\left(H_{1},H_{2},r\right)$ and $H_{k}=\;h\left(H_{1},H_{2}\right)⊕h\left(r,TMS_{pr}\right)$;
  - Then, the TMS stores the values ($H_{3},H_{k},r,T,TMS_{pb}$) into the smart card of $U_{i}$.
4. Login: The user $U_{i}$ must insert their identity $RL_{id}$ and secret password $RL_{pw}$ into the smart card reader during the login process. The smart card reader (SCR) performs the first level of authentication, as covered in the following steps.
  - The SCR calculates $H_{new1}=h\left(RL_{id},T,r\right)$ and $H_{new2}=h\left(RL_{pw},T,r\right)$;
  - If $H_{3}=h\left(H_{new1},H_{new2},r\right)$ is satisfied, then the login is accepted; otherwise, the login is denied;
  - Calculates $H_{k}⊕h\left(H_{1},H_{2}\right)=h\left(r,TMS_{pr}\right)$ and chooses (x) from $F_{d}$;
  - Calculates the equation Z=$\;x.TMS_{pb}$ and S=$\;h\left(h\left(r,T,TMS_{pr}\right),h\left(x.TMS_{pb}\right)\right)$;
  - Performs encryption for a random number x as $V=E_{TMS_{pb}}\left(x\right)$;
  - Generates a login request by using (V,S,T) and then send it to the TMS.
5. Mutual Authentication: If the TMS receives the login request triples (V,S,T), then it first checks the validity of the timestamp (T); if it is valid, then the TMS decrypts $\left(x\right)=D_{TMS_{pr}}\left(V\right)$. After this, the TMS calculates R =$\;h\left(h\left(r,T,TMS_{pr}\right),h\left(x.TMS_{pb}\right)\right)$ and compares the value of equality, i.e., R = S; if it is satisfied, then the server calculates K=$\;h\left(j.U_{pb}\right)$ and L= h(j.D), where j is a chosen number. Then, it sends (T,L) to$\;U_{i}$. When $U_{i}$ receives (L,T), it checks the validity of T and calculates K=$\;h\left(L.U_{pr}\right)$. Then, it passes the mutual authentication process and sets K as a secret key for communication. After, the user encrypts m as $C=E_{K}\left(m\right)$ and sends C to the TMS. When the TMS receives (C), it decrypts C as $m=D_{K}\left(C\right)$.
6. Password Update: This step is identical to the password update phase presented in [24].

## 5. Security Analysis

In this section, we conduct security analysis, including formal security analysis, based on the ROR model and informal security analysis based on mathematical assumptions. Both forms of analysis indicate that the proposed scheme is resistant to numerous cyber-attacks. The proposed two-factor authentication scheme’s security resilience is primarily based on a hyperelliptic curve discrete logarithm problem (HECDLP) and a one-way hash function. The HECDLP is the problem in which an adversary derives γ from the equation Q = γ.D. Given that the hash function is irreversible and resistant to collisions, it is reasonable to assume that this attempt will be challenging. The subsequent subsections elaborate on the security analysis.

### 5.1. Formal Security Analysis

We consider the adversary $\Gamma_{A}$ to possess complete control over the communication channel. Moreover, the participating devices are denoted by the symbols $I_{th}$ and $J_{th}$, whose instances are specified as $\omega =(\omega_{I}$,$\;\omega_{J}$). Therefore, $\Gamma_{A}$ may run the queries shown below.

Execute query: $\Gamma_{A}$ intercepts all the communicated messages that are transmitted between $(\omega_{I}$,$\;\omega_{J}$).

Send query: $\Gamma_{A}$ sends a message to ω and receives a response as a result.

Reveal query: $\Gamma_{A}$ is responsible for recovering the session key between $(\omega_{I}$,$\;\omega_{J}$).

Test query: $\Gamma_{A}$ ask for a session key from ω, and it returns a random bit $b_{It}$ in response.

Note that in the formal analysis, we consider h(.) as a random oracle that will be available for users and $\Gamma_{A}$. The following Theorem 1 is performed to demonstrate the session key security of the proposed scheme.

**Theorem** **1.** *Suppose* $\Gamma_{A}$ *makes the execution in a polynomial time (*$P_{tm}$*) to extract the session key that is suggested between* $\omega_{I}$ *and* $\omega_{J}$*. For the breaching probability, see Equation (1):*

(1) AΓA (Ptm)≤Qh2|Hash|+2. AΓA HECDLP(Ptm),

*where* $A_{\Gamma_{A}\;}{}^{HECDLP}$  *represents the non-ignorable advantages of* $\Gamma_{A}$ *to break the security of HECDLP,* |Hash| *denotes the range of*  h(.) *, and* $Qh^{2}$ *indicates the number of hash queries, respectively.*

**Proof of Theorem** **1:** We considered the following three Games $G_{i}$ (i = 1, 2, 3), and in each game, $\Gamma_{A}$, by using the test query, guesses a random bit $b_{It}$. Suppose $wins_{\Gamma_{A}}{}^{G_{i}}\;$is the event in which $\Gamma_{A}$ correctly guesses the bits $b_{It}$. The advantages of $\Gamma_{A}$ can be seen in Equation (2).

(2) $$A_{\Gamma_{A},G_{i}\;}\left(P_{tm}\right)=P𝓇\left(wins_{\Gamma_{A}}{}^{G_{i}}\right)$$

Game $G_{1}$: This game closely resembles the real scheme that is executed in the ROR model. In this game, we obtain the result as shown in Equation (3).

(3) $$A_{\Gamma_{A}\;}\left(P_{tm}\right)=|2.A_{\Gamma_{A},G_{i}\;}-1|$$

Game $G_{2}$: This game enables $\Gamma_{A}$ to intercept all the communicated messages between $\omega_{I}$ and $\omega_{J}$ and to extract the session key by using reveal and test queries to evaluate whether the generated key is random or real. $\Gamma_{A\;}$ intercepts (V,S,T), (T,L), and (C), where $V=E_{TMS_{pb}}\left(x\right),\;S$ = $h\left(h\left(r,T,TMS_{pr}\right),h\left(x.TMS_{pb}\right)\right)$, T is the public timestamp, and L= h(j.D), respectively. Hence, the session key can be obtained by $\Gamma_{A}$ if it is processed by equation K = $h\left(j.U_{pb}\right)$ or K=$\;h\left(L.U_{pr}\right)$, in which we will first find *j* and $U_{pr}$, two unknown variables; thus, it shows that,$\;\Gamma_{A}$ has negligible probability and $G_{1},G_{2}$ are indistinguishable, as shown in Equation (4).

(4) $$A_{\Gamma_{A},G_{1}\;}=A_{\Gamma_{A},G_{2}\;}$$

Game $G_{3}$: This game includes the send and hash queries. As we know, in $G_{2}$, all the intercepted messages (V,S,T), (*T*, *L*), and (C) have no positive results because all the messages are safeguarded through HECDLP and a one-way hash function. So, we can say that $G_{1},G_{2}$ are indistinguishable, determine the advantages of breaking HECDLP of $A_{\Gamma_{A}\;}{}^{HECDLP}\left(P_{tm}\right),$ and, by utilizing all the hash queries, we can obtain the equation Qh22.|Hash|. Generally, the following outcomes are achieved, shown in Equation (5):

(5) AΓA,G2 −AΓA,G3 ≤Qh22.|Hash|+ AΓA HECDLP(Ptm).

Hence, $\Gamma_{A}$ can execute all the queries and guess the bits $b_{It}$, so the following outcomes can be received, shown in Equation (6):

(6) AΓA,G3 ≤12.

From Equations (3) and (4), the following results can be derived:

(7) 12. AΓA HECDLP(Ptm)=|AΓA,G1 −12|=|AΓA,G2 −12|.

From Equations (6) and (7), we can obtain the following outcomes:

(8) 12. AΓA HECDLP(Ptm)=|AΓA,G2 −AΓA,G3 |.

From Equations (5) and (8), we can obtain the following outcomes:

(9) 12. AΓA HECDLP(Ptm)=Qh22.|Hash|+ AΓA HECDLP(Ptm).

Multiplying 2 by both sides of Equation (9) yields the following results:

AΓA HECDLP(Ptm)=Qh2|Hash|+ 2.AΓA HECDLP(Ptm), hence, it is proved. □

### 5.2. Informal Security Analysis

In this subsection, we used the mathematical assumptions of the hash function and HECDLP to do the following informal analysis.

#### 5.2.1. Confidentiality

This scheme property can be followed if there are no attackers that can steal the contents of C. The attacker can first try to produce the secret key from equation K = $h\left(j.U_{pb}\right)$ or K =$\;h\left(L.U_{pr}\right)$. The equation $K=\;h\left(j.U_{pb}\right)$ contains the private number j that belongs to the finite field of the hyperelliptic curve ($F_{d}$), and this is only known to the TMS and the public key of the user $U_{pb}$. For the attacker to obtain j, they must solve the hyperelliptic curve discrete logarithm problem, which is impossible for them. The other equation, K = $h\left(L.U_{pr}\right)$, contains the public number L and the private key of the user. The user’s private key is only known to the user, and if an attacker wishes to obtain access to the private key used to generate the secret key, the attacker must solve the hyperelliptic curve discrete logarithm problem, which consists of an equation $U_{pb}$ = $U_{pr}.D$.

#### 5.2.2. Integrity

If no attacker can modify the contents of m, this property can be obeyed in the scheme. In the proposed scheme, the user can encrypt m as $C=E_{K}\left(m\right)$ and send C and A=h(m) to the TMS. When the TMS receives (C,A), it can decrypt C as $m=D_{K}\left(C\right)$, calculate B=h(m), and compare B=A; if the condition is met, there are no modifications to the message. The first thing the attacker can do is attempt to derive the secret key from the equation K = $h\left(j.U_{pb}\right)$ or K = $h\left(L.U_{pr}\right)$, depending on which one they prefer. The private number j, which belongs to the finite field of the hyperelliptic curve ($F_{d}$), is included in the equation K = $h\left(j.U_{pb}\right)$. This value is only known to the TMS, together with the public key of the user $U_{pb}$. In this case, for the attacker to obtain *j*, because of this, they can pass through the hyperelliptic curve discrete logarithm problem, which would otherwise be impossible for them here. If an attacker wants to access a specific private key that is used for the generation of a secret key, then it must solve the hyperelliptic curve discrete logarithm problem because it is made up of the equation $U_{pb}$ = $U_{pr}.D$. Another equation, K = $h\left(L.U_{pr}\right)$, contains the public number L and the private key of the user, so the private key of the user is only known to that user. The second factor is that the message is secure by an irreversible one-way hash algorithm, so the attacker cannot alter it.

#### 5.2.3. Forward Security

This scheme fulfils the need for forward security because it does not directly use the private key of the server or user for the encryption and decryption of a message. The process for the encryption of the message in the proposed scheme allows the user to encrypt m as $C=E_{K}\left(m\right)$ and send C and A=h(m) to the TMS. When the TMS receives (*C*, *A*), it can decrypt C as $m=D_{K}\left(C\right)$, compute B=h(m), and compare B=A; if it is satisfied, then there are no modifications in the message. The proposed scheme ensures forward security by not reusing the same private key for each session and by renewing the secret key for each new session.

#### 5.2.4. Anonymity and Untraceability

For the formation of the login request, the user must complete the following actions:

- Calculate $H_{new1}=h\left(RL_{id},T,r\right)$ and $H_{new2}=h\left(RL_{pw},T,r\right)$;
- Verify if $H_{3}=h(H_{new1},H_{new2},r$) is satisfied; if yes, the login will be authorized; otherwise, the login will be denied;
- Calculate $H_{k}⊕h\left(H_{1},H_{2}\right)=h\left(r,TMS_{pr}\right)$ and choose (x) from $F_{d}$;
- Calculate the equation Z = $x.TMS_{pb}$ and S = $h\left(h\left(r,T,TMS_{pr}\right),h\left(x.TMS_{pb}\right)\right)$;
- Perform encryption for a random number x as $V=E_{TMS_{pb}}\left(x\right)$;
- Generate a login request by using (V,S,T) and sends it to the TMS.

The triples (V,S,T) do not include any user identifiers; hence, we must conclude that the proposed scheme satisfies the anonymity and untraceability requirements.

#### 5.2.5. Resist against Replay Attack

The user *U_i_* must insert their identity $RL_{id}$ and secret password $RL_{pw}$ into the smart card reader during the login process. The SCR performs the first level of authentication, detailed in the following steps.

- Calculates $H_{new1}=h\left(RL_{id},T,r\right)$ and $H_{new2}=h\left(RL_{pw},T,r\right)$;
- Checks if $H_{3}=h\left(H_{new1},H_{new2},r\right)$ is satisfied; if yes, then the login will be permitted; otherwise, it rejects the login;
- Computes $H_{k}⊕h\left(H_{1},H_{2}\right)=h\left(r,TMS_{pr}\right)$ and chooses (x) from $F_{d}$;
- Compute the equations Z = $x.TMS_{pb}$ and S = $h\left(h\left(r,T,TMS_{pr}\right),h\left(x.TMS_{pb}\right)\right)$;
- Does encryption for a random number x as *V* = *E_(TMS_pb_)_*(*X*);
- Generates a login request by using (V,S,T) and sends it to the TMS.

If the TMS receives the login request triples (V,S,T), then it first authenticates the validity of timestamp (T); if it is valid, then the TMS decrypts $\left(x\right)=D_{TMS_{pr}}\left(V\right)$. After, the TMS calculates R = $h\left(h\left(r,T,TMS_{pr}\right),h\left(x.TMS_{pb}\right)\right)$ and compares values such as the equality of R = S; if it is satisfied, then the server computes K = $h\left(j.U_{pb}\right)$ and L = h(j.D), where j is chosen number. Then, it sends (T,L) to $U_{i}$. When $U_{i}$ receives (L,T), it checks the validity of T and computes K = $h\left(L.U_{pr}\right)$; then, it passes the mutual authentication process and sets K as the secret key for communication. After, the user can encrypt m as $C=E_{K}\left(m\right)$ and send C and A=h(m) to the TMS. When the TMS receives (C,A), it decrypts C as $m=D_{K}\left(C\right)$, computes B=h(m), and compares B=A; If fulfilled, it demonstrates that the message has not been modified. We can conclude from these communication processes that the proposed scheme is resistant to replay attacks due to the use of a new time stamp for each transmitted message.

#### 5.2.6. Resistant against Denial-of-Service Attacks

The proposed scheme will be secured from denial-of-service (DoS) attacks using the following steps: When the TMS receives the login request triples (V,S,T), it first checks the validity of timestamp (T); if it is valid, then the TMS decrypts $\left(x\right)=D_{TMS_{pr}}\left(V\right)$. After, the TMS computes R = $h\left(h\left(r,T,TMS_{pr}\right),h\left(x.TMS_{pb}\right)\right)$ and compares values such as the equality of R = S; if it is satisfied, then the server computes K = $h\left(j.U_{pb}\right)$ and L = h(j.D), where j is a chosen number. Then, it sends (T,L) to $U_{i}$. When $U_{i}$ receives (L,T), it first checks the validity of T and computes K = $h\left(L.U_{pr}\right)$; then, it passes the mutual authentication process and sets K as a secret key for communication that indicates that the proposed scheme is resistant to DoS attacks since every new user must undergo the above-mentioned authentication processes.

#### 5.2.7. Mutual Authentication

The proposed scheme provides mutual authentication using the following steps: When the TMS receives the login request triples (V,S,T), it first checks the validity of timestamp (*T*); if it is valid, then the TMS decrypts$\;\left(x\right)=D_{TMS_{pr}}\left(V\right)$. After, the TMS computes R = $h\left(h\left(r,T,TMS_{pr}\right),h\left(x.TMS_{pb}\right)\right)$ and compares values such as the equality of R = S; if it is satisfied, then the server computes K = $h\left(j.U_{pb}\right)$ and L = h(j.D), where j is a chosen number. Then, it sends (T,L) to $U_{i}$. When $U_{i}$ receives (L,T), it first checks the validity of T, computes K = $h\left(L.U_{pr}\right)$, and passes the mutual authentication process, with K set as a secret key for communication. It means that the user and the TMS can mutually authenticate each other in this way.

#### 5.2.8. Key Agreement

The proposed scheme provides key agreement while using the following steps: When the TMS receives the login request triples (V,S,T), it first checks the validity of timestamp (T); if it is valid, then the TMS decrypts $\left(x\right)=D_{TMS_{pr}}\left(V\right)$. After, the TMS computes R = $h\left(h\left(r,T,TMS_{pr}\right),h\left(x.TMS_{pb}\right)\right)$ and compares values such as the equality of R = S; if it satisfied, then server computes K = $h\left(j.U_{pb}\right)$ and L = h(j.D), where j is a chosen number. It sends (T,L) to $U_{i}$. When $U_{i}$ receives (L,T), it checks the validity of T and computes K = $h\left(L.U_{pr}\right)$. After these steps, it passes the mutual authentication process and sets K as a secret key for communication. After, the user can encrypt m as $C=E_{K}\left(m\right)$ and send C and A=h(m) to the TMS. When the TMS receives (C,A), it can decrypt C as $m=D_{K}\left(C\right)$, compute B=h(m), and compare B=A; if it is satisfied, it indicates that the message has not been altered.

## 6. Performance Analysis

This analysis evaluates the proposed scheme’s performance based on its computation and communication costs, which is accomplished by comparing the proposed scheme to equivalent existing schemes.

### 6.1. Computational Cost

The analysis of computation cost between the proposed scheme and those proposed by Qiu et al. [21], Ostad-Sharif et al. [22], and Radhakrishnan et al. [24] considers the major operations involved in a cryptographic scheme, including the hash function elliptic curve addition, elliptic curve multiplications, hyperelliptic curve addition, and elliptic curve addition. For this, we used the symbols $TM_{h},TM_{em}$, and $TM_{hm}$ to denote a single operation of the hash function, elliptic curve addition, elliptic curve multiplications, hyperelliptic curve addition, and elliptic curve addition. Table 2 compares the costs of computation. In addition, for a more precise understanding, we have included the performance study comparisons of the proposed scheme to the other schemes in terms of computation cost per millisecond. The time in milliseconds for the major operations, such as $TM_{h},TM_{em}$, are adopted from Yu et al. [25]’s scheme, wherein the authors considered the following hardware and software as part of an experimental setup:

- The CPU architecture is 64 bits, and the processor is an Intel Core i5-10400 running at 2.90 GHz with six cores; there is also 16 GB of RAM;
- Operating System: Ubuntu 18.04 LTS;
- Library: MIRACL [26].

There have been 100 runs for each primitive. For each primitive, the maximum and minimum timings in milliseconds are noted. In addition, the average running time (in milliseconds) over these 100 runs is calculated concurrently. The maximum time consumed by $TM_{h}$ and $TM_{em}$ is 0.149 and 2.737, respectively. The minimum time consumed by $TM_{h}$ and $TM_{em}$ is 0.024 and 0.472, respectively. The average time consumed by $TM_{h}$ and $TM_{em}$ is 0.055 and 0.522, respectively. We assume half time for the hyperelliptic curve relative to the elliptic curve and consider the maximum, minimum, and average time as 1.3685, 0.236, and 0.261, respectively, as the hyperelliptic curve typically consumes half the time of the elliptic curve [27,28]. So, in the following steps, we compared our scheme with Qiu et al. [21], Ostad-Sharif et al. [22], and Radhakrishnan et al. [24], considering the maximum, minimum, and average time in milliseconds.

- Maximum time in milliseconds: considering the maximum time, Qiu et al. [21] need $8TM_{h}+2TM_{em}=8\times 0.149+2\times 2.737=6.666$ at sender side, $5TM_{h}+2TM_{em}=5\times 0.149+2\times 2.737=6.219$ at the receiver side, and the total is $13TM_{h}+4TM_{em}=13\times 0.149+4\times 2.737=12.885$; Ostad-Sharif et al. [22] need $7TM_{h}+2TM_{em}=7\times 0.149+2\times 2.737=6.517$ at sender side, $7TM_{h}+2TM_{em}=7\times 0.149+2\times 2.737=6.517$ at the receiver side, and the total is $14TM_{h}+4TM_{em}=14\times 0.149+4\times 2.737=13.034$; Radhakrishnan et al. [24] need $10TM_{h}+3TM_{em}=10\times 0.149+3\times 2.737=9.701$ at sender side, $3TM_{h}+3TM_{em}=3\times 0.149+3\times 2.737=8.653$ at the receiver side, and the total is $13TM_{h}+6TM_{em}=13\times 0.149+6\times 2.737=18.354$; and our proposed scheme needs $10TM_{h}+3TM_{hm}=10\times 0.149+3\times 1.3685=5.5955$ at sender side, $3TM_{h}+3TM_{hm}=3\times 0.149+3\times 1.3685=4.5525$ at the receiver side, and the total is $13TM_{h}+3TM_{hm}=13\times 0.149+6\times 1.3685=10.148$
- Minimum time in milliseconds: for the minimum time in milliseconds, Qiu et al. [21] need $8TM_{h}+2TM_{em}=8\times 0.024\;+2\times 0.472=1.136$ at sender side, $5TM_{h}+2TM_{em}=5\times 0.024\;+2\times 0.472=1.064$ at the receiver side, and the total is $13TM_{h}+4TM_{em}=13\times 0.024+4\times 0.472=2.2;$ Ostad-Sharif et al. [22] need $7TM_{h}+2TM_{em}=7\times 0.024\;+2\times 0.472=1.112$ at sender side, $7TM_{h}+2TM_{em}=7\times 0.024\;+2\times 0.472=1.112$ at the receiver side, and the total is $14TM_{h}+4TM_{em}=14\times 0.024\;+4\times 0.472=2.224$; Radhakrishnan et al. [24] need $10TM_{h}+3TM_{em}=10\times 0.024+3\times 0.472=1.656$ at sender side, $3TM_{h}+3TM_{em}=3\times 0.024\;+3\times 0.472=1.488$ at the receiver side, and the total is $13TM_{h}+6TM_{em}=13\times 0.024\;+6\times 0.472=3.144$; and our proposed scheme needs $10TM_{h}+3TM_{hm}=10\times 0.024\;+3\times 0.236=0.948$ at sender side, $3TM_{h}+3TM_{hm}=3\times 0.024\;+3\times 0.236=0.78$ at the receiver side, and the total is $13TM_{h}+3TM_{hm}=13\times 0.024+6\times 0.236=1.728.$
- Average time in milliseconds: for the average time in milliseconds, Qiu et al. [21] need $8TM_{h}+2TM_{em}=8\times 0.055\;+2\times 0.522=1.484$ at sender side, $5TM_{h}+2TM_{em}=5\times 0.055\;+2\times 0.522=1.319$ at the receiver side, and the total is $13TM_{h}+4TM_{em}=13\times 0.055\;+4\times 0.522=2.803;$ Ostad-Sharif et al. [22] need $7TM_{h}+2TM_{em}=7\times 0.055\;+2\times 0.522=1.429$ at sender side, $7TM_{h}+2TM_{em}=7\times 0.055\;+2\times 0.522=1.429$ at the receiver side, and the total is $14TM_{h}+4TM_{em}=14\times 0.055\;+4\times 0.522=2.858$; Radhakrishnan et al. [24] need $10TM_{h}+3TM_{em}=10\times 0.055\;+3\times 0.522=2.116$ at sender side, $3TM_{h}+3TM_{em}=3\times 0.055\;+3\times 0.522=1.721$ at the receiver side, and the total is $13TM_{h}+6TM_{em}=13\times 0.055\;+6\times 0.522=3.837$; and our proposed scheme needs $10TM_{h}+3TM_{hm}=10\times 0.055\;+3\times 0.261=1.333$ at sender side, $3TM_{h}+3TM_{hm}=3\times 0.055\;+3\times 0.261=0.948$ at the receiver side, and the total is $13TM_{h}+3TM_{hm}=13\times 0.055+6\times 0.261=2.281.$

**Figure 2:**
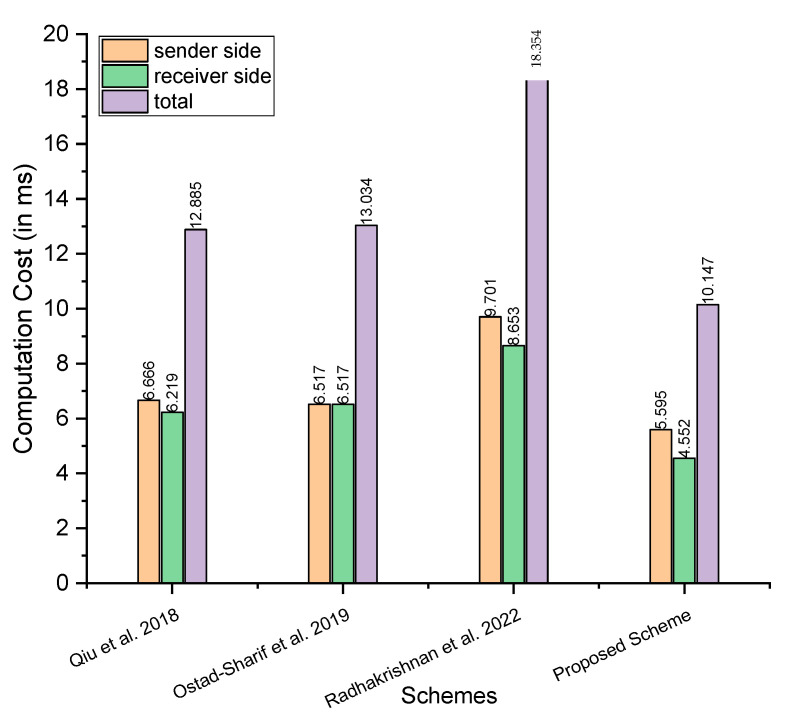
Comparison of computation costs (the maximum time in ms) [21,22,24].

**Figure 3:**
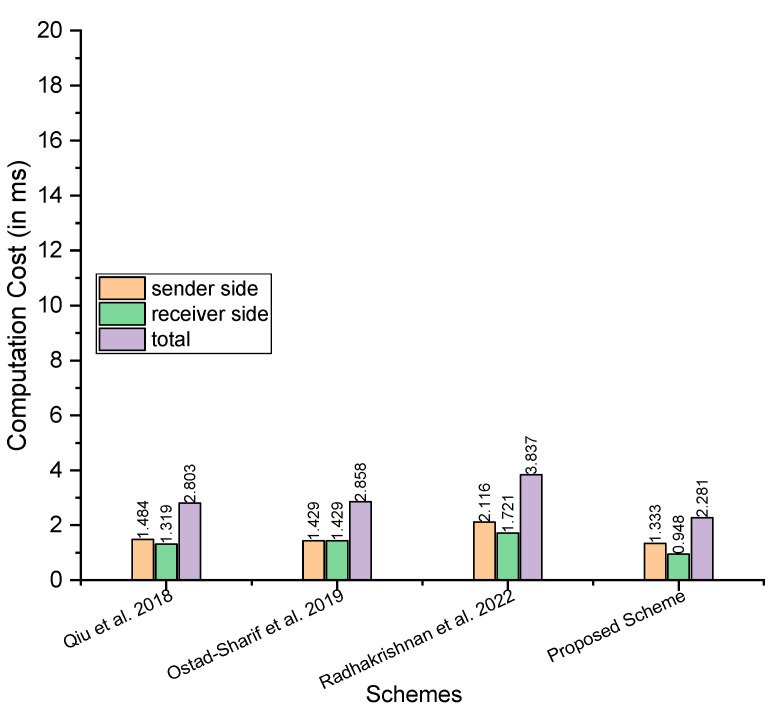
Comparison of computation costs (the average time in ms) [21,22,24].

**Figure 4:**
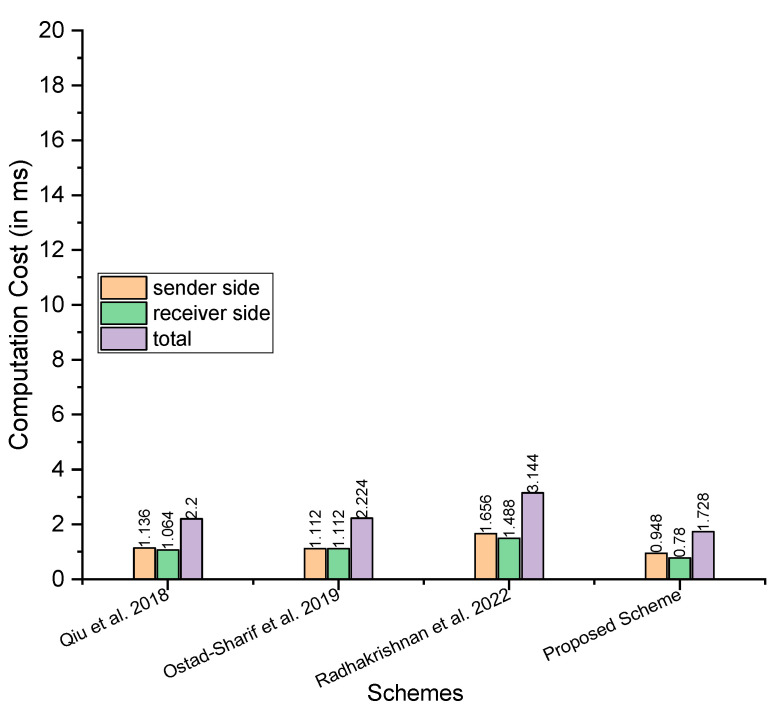
Comparison of computation costs (the minimum time in ms) [21,22,24].

Figure 2, Figure 3 and Figure 4 depict a comparison of computation costs based on maximum, average and minimum time in milliseconds, demonstrating the better efficiency of the proposed method in terms of computation costs.

### 6.2. Communication Cost

**Figure 5:**
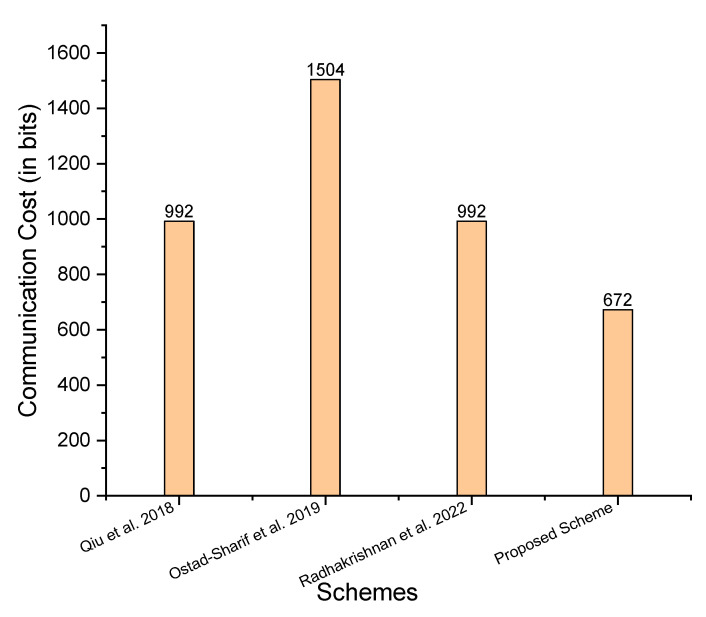
Comparison of communication costs (in bits) [21,22,24].

Communication cost refers to the number of bits sent during the transmission session in addition to the ciphertext or message. When calculating the communication cost, extra bits are typically counted as elliptic curve parameter size, hyperelliptic curve parameter size, and bilinear pairing parameter size. Table 2 provides a comparison of the communication cost between the schemes proposed by Qiu et al. [21], Ostad-Sharif et al. [22], and Radhakrishnan et al. [24], based on the major operations. Table 2 and Figure 5 show a comparison of communication costs in bits, which reveals that the proposed scheme has lower communication costs.

## 7. Conclusions

Since the TMIS utilizes the Internet to connect biomedical equipment and sensors, this system is vulnerable to a wide range of cryptographic attacks. Several researchers have proposed smart card-based mutual authentication schemes to prevent cryptographic assaults in the available literature. However, such solutions were frequently implemented using computationally expensive procedures, such as bilinear pairing, elliptic curve operations, etc., which were inappropriate for biomedical apparatus and sensors that typically have limited computational resources. In this article, we proposed a two-factor mutual authentication scheme utilizing smart cards and HECC. This new scheme utilized the finest characteristics of HECC, including compact parameters and key sizes, to enhance the real-time performance of an IoT-based TMIS system. A comprehensive formal and informal security analysis demonstrated that the proposed scheme is resistant to a wide variety of cryptographic attacks. In addition, a comparison of computation and communication costs revealed that the proposed scheme requires less computation and communication costs than similar available schemes.

## Figures and Tables

**Table 1:** Notation’s table.

S. No	Symbol	Description
1	h	The hash function that belongs to the SHA family
2	$TMS_{pr}$	The private key of the TMS, which is selected from the finite field ($F_{d}$)
3	($F_{d}$)	The finite field of a hyperelliptic curve has a size that is not greater than 80 bits
4	($HEC_{g=2}$)	The hyperelliptic curve with genus equal to or less than 2
5	(D)	The devisor is on a hyperelliptic curve and has a size that is not greater than 80 bits
6	$RL_{id}$	The real identity of the user $U_{i}$
7	($RL_{pw}$)	The password for the user $U_{i}$
8	$TMS_{pb}$	The public key of the TMS which is the multiplication of the private key $TMS_{pr}\;\mathrm{and}\;\mathrm{devisor}\;\left(D\right)$
9	$E_{TMS_{pb}}$	Encryption process by using the public key of the TMS
10	⊕	Used to represent the encryption and decryption
11	$D_{TMS_{pr}}$	Decryption process by using the private key of the TMS
12	T	It is used to represent the timestamp
13	x,r,j	Three randomly generated numbers from ($F_{d}$)
14	K	The secret key which is shared among the TMS and the user
15	$E_{K}$	Encryption process by using the shared secret key of the TMS and the user
16	$D_{K}$	Decryption process by using the shared secret key of the TMS and the user

**Table 2:** Communication costs comparison (in bits).

Schemes	Communication Cost	Communication Cost in Bits
Qiu et al. [21]	$2BM_{h}+3BM_{em}$	992
Ostad-Sharif et al. [22]	$4BM_{h}+3BM_{em}$	1504
Radhakrishnan et al. [24]	$2BM_{h}+3BM_{em}$	992
Proposed Scheme	$2BM_{h}+2BM_{hm}$	672

Note: Symbols $BM_{h}$,$\;BM_{em}$, and $BM_{hm}$, represent the extra bits of the hash function, elliptic curve parameter, and hyperelliptic curve parameter, respectively. We assume $BM_{h}=256\;\mathrm{bits}$, $BM_{em}=160\;\mathrm{bits}$, and $BM_{hm}=80\;\mathrm{bits}$.

## Data Availability

Not applicable.

## Abbreviations

The following acronyms and initalisms are used in this manuscript.

BP	bilinear pairing
DOS	denial of service
ECC	elliptic curve cryptography
HECC	hyperelliptic curve cryptography
HECDLP	hyperelliptic curve discrete logarithm problem
IoT	Internet of things
KGC	key generation centre
MIRACL	multiprecision integer and rational arithmetic cryptographic
ROM	random oracle mode
ROR	real-or-random
RSA	Rivest-Shamir-Adleman
SHA	secure hashing algorithm
TMIS	telecare medicine information system
TMS	telemedicine server
